# Lifestyle and health status in a sample of Swedish women four years after pregnancy: a comparison of women with a history of normal pregnancy and women with a history of gestational diabetes mellitus

**DOI:** 10.1186/s12884-015-0487-2

**Published:** 2015-03-13

**Authors:** Margareta Persson, Anna Winkvist, Ingrid Mogren

**Affiliations:** School of Health and Social Studies, Dalarna University, Falun, Sweden; Department of Nursing, Umeå University, SE – 901 87 Umeå, Sweden; Department of Internal Medicine and Clinical Nutrition, Sahlgrenska Academy, University of Gothenburg, Gothenburg, Sweden; Department of Clinical Sciences, Obstetrics and Gynecology, Umeå University, Umeå, Sweden

## Abstract

**Background:**

Despite the recommendations to continue the regime of healthy food and physical activity (PA) postpartum for women with previous gestational diabetes mellitus (GDM), the scientific evidence reveals that these recommendations may not be complied to. This study compared lifestyle and health status in women whose pregnancy was complicated by GDM with women who had a normal pregnancy and delivery.

**Methods:**

The inclusion criteria were women with GDM (ICD-10: O24.4 A and O24.4B) and women with uncomplicated pregnancy and delivery in 2005 (ICD-10: O80.0). A random sample of women fulfilling the criteria (n = 882) were identified from the Swedish Medical Birth Register. A questionnaire was sent by mail to eligible women approximately four years after the pregnancy. A total of 444 women (50.8%) agreed to participate, 111 diagnosed with GDM in their pregnancy and 333 with normal pregnancy/delivery.

**Results:**

Women with previous GDM were significantly older, reported higher body weight and less PA before the index pregnancy. No major differences between the groups were noticed regarding lifestyle at the follow-up. Overall, few participants fulfilled the national recommendations of PA and diet. At the follow-up, 19 participants had developed diabetes, all with previous GDM. Women with previous GDM reported significantly poorer self-rated health (SRH), higher level of sick-leave and more often using medication on regular basis. However, a history of GDM or having overt diabetes mellitus showed no association with poorer SRH in the multivariate analysis. Irregular eating habits, no regular PA, overweight/obesity, and regular use of medication were associated with poorer SRH in all participants.

**Conclusions:**

Suboptimal levels of PA, and fruit and vegetable consumption were found in a sample of women with a history of GDM as well as for women with normal pregnancy approximately four years after index pregnancy. Women with previous GDM seem to increase their PA after childbirth, but still they perform their PA at lower intensity than women with a history of normal pregnancy. Having GDM at index pregnancy or being diagnosed with overt diabetes mellitus at follow-up did not demonstrate associations with poorer SRH four years after delivery.

**Electronic supplementary material:**

The online version of this article (doi:10.1186/s12884-015-0487-2) contains supplementary material, which is available to authorized users.

## Background

Internationally, gestational diabetes mellitus (GDM) affects a rising number of pregnant women in most ethnic groups [1]. Groups of women of different ethical origins present different prevalences of GDM, where Caucasians show the lowest and Asians the highest prevalence of GDM [2]. In a Swedish study, 44.5% of pregnant women with GDM are of non-Nordic origin, even though 25.3% of all women of fertile age are of non-Nordic origin in the population of the study area [3]. Further, the prevalence of GDM is doubled in overweight and is increased six-fold in obese women [4]. The risk of adverse pregnancy and delivery outcomes is increased for women with GDM. Even women with an oral glucose tolerance test below the diagnostic cut-off for GDM have increased risks of adverse pregnancy and delivery outcomes [5].

Within the first five to ten years after a pregnancy complicated by GDM, the cumulative incidence of type 2 diabetes rapidly increases [6]. The risk of developing type 2 diabetes is more than seven-fold for women with previous GDM compared to women with normoglycemic pregnancies [7].

According to a Cochrane review [8], interventions of either diet or exercise alone do not affect the incidence of type 2 diabetes in high-risk populations. Combined diet-and exercise intervention has demonstrated positive effects on blood pressure, body weight, and waist circumference, and also a decrease of the incidence of type 2 diabetes by 37% [8].

Women with previous GDM are recommended lifestyle interventions to prevent overt diabetes mellitus [9]. However, more women gain weight than lose weight postpartum, despite their risk of developing overt diabetes [10]. Perceived barriers to maintain healthy eating and physical activity (PA) postpartum include constraints related to time, finances, child care, work, motivation and fatigue [11].

Lifestyle and health status after pregnancy with GDM, have not previously been studied in a Swedish setting. As shown by Berg et al. [3], a larger proportion of non-Nordic born pregnant women develop GDM, hence comparing the life style and health status after pregnancy for women born in or born outside Sweden would be of interest. This study compared lifestyle and health status approximately four years after childbirth in a random selected sample of women whose pregnancy was complicated by GDM with women who had a normal pregnancy. The specific aims were: 1) to investigate differences in lifestyle; 2) to investigate differences in health outcomes; and 3) to investigate the associations between lifestyle and health outcomes.

## Methods

This retrospective study was part of a larger research project investigating health status, quality of life, wellbeing, and lifestyle after normal pregnancy and pregnancy complicated with GDM in Sweden. The study was approved by the Regional Ethical Review Board at Umeå University (Dno: 05-020 M).

### Data from the Swedish medical birth register

The Swedish Medical Birth Register (MBR) was established in 1973 and data are collected on almost all pregnancies and deliveries. As it is mandatory for health service providers to report data to the MBR, data for approximately 100 000 deliveries are entered annually [12]. Initially in this project, medical data were retrieved from the MBR by statisticians at the National Board of Health and Welfare, representing a national randomly selected sample of women with delivery during 2005. The sample consisted of 120 women with diet-treated GDM (ICD-10 code: O24.4A), and 120 women with insulin-treated GDM (ICD-10 code O24.4B), and 650 women with normal pregnancy and birth (ICD-10 code: O80.0 and no additional codes), comprising a total sample of 890 women.

The size of the estimated sample (n = 890) was based on the assumption that the prevalence of later poor self-rated health (SRH) would be doubled in women with a history of GDM compared to women with normal pregnancies. Accordingly, we calculated that at least 84 women with a history of GDM (diet or insulin treatment) and 560 women with normal pregnancy would be needed to demonstrate a difference in later poorer SRH at a significance level of 0.05. Presuming a dropout rate of approximately 20% for the follow-up study, a total of 890 women would be necessary to address the issue of SRH.

### Procedure, questionnaire and data collection

**Figure 1:**
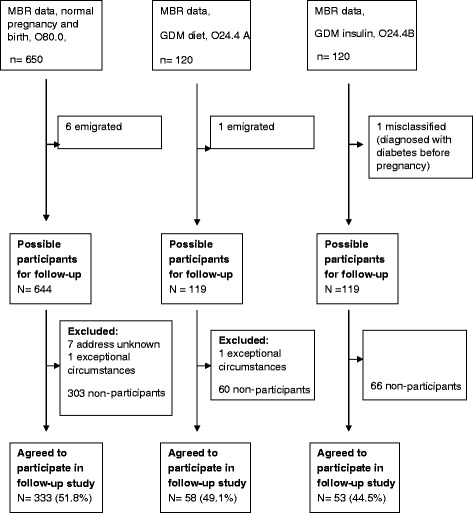
**Recruitment procedures of participants in follow-up study.**

A flow chart of the complete recruitment procedure is presented in Figure 1. Between May and June 2009, 882 eligible women from the initial sample obtained from the MBR, were mailed an invitation to participate in a questionnaire follow-up study (approximately four years after the index pregnancy). The mail included information about the study, a consent form, a questionnaire and a prepaid envelope. One reminder containing the same material as the first invitation was mailed if needed.

The research team constructed a questionnaire that included quality of life (standard SF-36 form [13-15]), and items regarding medical history, self-reported aspects of dietary intake, and PA. Before data collection, a small pilot study of ten purposively selected women, who recently had given birth, was performed to test the questionnaire. This pilot study resulted in minor changes of the design of the questionnaire and rephrasing of a few questions. In this paper, questionnaire data addressing issues related to lifestyle and health status will be presented.

### Variable definitions and processing of data

#### Self-rated health (SRH)

The respondents were asked to rate their present health as excellent, very good, good, fair or poor. The individual perception of health has shown associations with future mortality. A meta-analysis of 22 studies show that individuals reporting poor SRH on a simple general SRH item have a two-fold increased risk of mortality compared to individuals reporting excellent health [16]. For the analysis, this outcome was categorized as excellent/very good/good (i.e. good health) or fair/poor (i.e. poorer health).

#### Recommendations of daily intake of vegetables and fruits

The study used the National Food Agency (NFA) recommendation of a daily consumption of 500 g of vegetables and fruits, which is equivalent to three fruits and two large servings of vegetables [17]. In the questionnaire, this information was included as an example of the amounts required to fulfill the recommendation to 100%. The respondents indicated to what extent they fulfilled the recommendation on an “ordinary” day on a 100 mm VAS scale with anchors 0% and 100% of the recommended levels. All responses on the VAS scale were manually measured; for example a value of 60 mm was equivalent to 60%.

#### Compliance to received dietary advice

The women with a history of GDM responded to statements on how they perceived and adhered to the dietary advice received during and after pregnancy. The statements were for example: “To what extent did you find the dietary advice useful for your situation during pregnancy” and “To what extent did you find the dietary advice important for your situation after child birth”. The answers were given on a 100 mm VAS scale with anchors indicating 0% (not at all useful/important) and 100% (very useful/important). All responses were manually measured in the same manner as described above.

#### Physical activity (PA)

The Swedish national recommendations of PA during pregnancy suggest a minimum of 30 minutes of moderate intensity daily [18]. Respondents were asked to estimate the intensity of their PA (low intensity: able to speak/no heavy breathing during performance; moderate intensity: able to speak with some effort/some heavy breathing; and high intensity: not able to speak/ heavy breathing during performance). The NFA has suggested that this categorization of the intensity of PA is appropriate, when evaluating PA in population studies [19]. In addition, the participants were asked to estimate the frequency of their PA (per week) before pregnancy, during pregnancy and during the six months before the survey. If a respondent answered 3 to 4 times a week, this was calculated as 3.5 times a week for the purpose of the analysis.

The total number of participants with GDM diagnosis (diet-treated and insulin-treated GDM, n = 111) was small: 52.3% (n = 58) were treated with a combination of diet and physical exercise and 47.7% (n = 53) received insulin therapy at the index pregnancy. As no significant differences were found comparing responses of health status or lifestyle, the subgroups were merged when comparing the outcomes to those of the normal pregnancy group.

Further, available data from the MBR were used to compare characteristics of participants and non-participants.

### Statistical analysis

An overview of outcomes and potential predictors is presented in Additional file 1. Categorical variables were analyzed using Pearson’s Chi^2^-test and Fisher’s exact test when appropriate. The continuous variables were compared using Student’s *t*-test at normal distributions and Mann–Whitney *U*-test when the distribution was skewed, and standard deviation and 25^th^ – 75^th^ percentile are presented respectively. For the analysis of present lifestyle and health status, sixteen women were excluded from the sample. Fourteen women were pregnant and two women had not stated whether they were pregnant or not at the time of the follow-up. This exclusion of these participants was made as pregnancy may contribute to alterations in lifestyle as well as health status. Four of the 14 pregnant women had a history of GDM in their previous pregnancy in 2005; however none of these women had developed overt diabetes mellitus before the current pregnancy.

Univariate logistic regression analyses were used to evaluate the associations between outcomes and possible predictors. Multiple logistic regression analyses were performed by entering predictors found to be significant in the univariate analysis in a stepwise manner. Only the results of the last step of this stepwise analysis are presented in the results section. Significance level was set at p < 0.05. Calculations were performed using SPSS for Windows, version 20.0 (IBM, Somers, NY).

To clarify the time period addressed when presenting the findings, the time period will be indicated by using “prior to index pregnancy” to indicate patients’ status before index pregnancy, and “during index pregnancy” to indicate patients’ status during pregnancy. The patients’ status at the follow-up is labelled “present status”.

## Results

### Characteristics of participants

A sample of 444 women (50.8% of all eligible women) agreed to participate in the follow-up study approximately four years after the index pregnancy. Characteristics of the participants and a comparison of characteristics of participants born in and outside of Sweden are presented in Table 1. The women with a history of normal pregnancy (n = 333) were younger, more often born in Sweden, more often reported normal BMI (18.50 – 24.99 kg/m^2^), and were more physically active before their index pregnancy than women with a history of GDM (n = 111). Women with normal pregnancies were taller (p < 0.001) and had lower body weight (p = 0.001); a mean of 1.67 m (SD ± 0.07) and a median body weight of 63.0 kg (25th – 75th percentile: 57.0 – 72.0 kg) respectively compared to women with a history of GDM. These women had a mean maternal height of 1.65 m (SD ± 0.06) and a median body weight of 69.5 kg (25th – 75th percentile: 58.0 – 82.0 kg). However, women with a history of GDM reported less weight gain during their pregnancy compared to women with normal pregnancy. Despite regular PA as part of the treatment of GDM, 36.1% of women with a history of GDM reported performing PA on a regular basis during pregnancy. No significant differences between women with normal pregnancy and women with GDM-affected pregnancy were seen regarding number of pregnancies, number of children born, or marital status. A total of 12.7% of all participants were born outside Sweden; 50.9% from countries in Asia, 30.9% from European countries, 10.9% originated from South America and 7.3% from African countries.

**Table 1:** **Characteristics of participants at index pregnancy 2005 presented as women with normal pregnancy and women with pregnancy complicated by gestational diabetes mellitus (GDM) and as born in and outside Sweden**

	**Normal pregnancy**	**Pregnancy with GDM**	**P-value**	**Participants born in Sweden with normal pregnancy**	**Participants born in Sweden with pregnancy with GDM**	**P-value**	**Participants born outside Sweden with normal pregnancy**	**Participants born outside Sweden with pregnancy with GDM**	**P-value**
**Status prior to index pregnancy 2005**	**(N = 333)**	**(N = 111)**		**(N = 303)**	**(N = 83)**		**(N = 29)**	**(N = 27)**	
**PCOS diagnosis**	5 (1.5%)	8 (7.6%)	0.004 1	4 (1.3%)	6 (7.4%)	0.008^1^	0 (0%)	2 (8.7%)	0.198^1^
**Infertility treatment**	17 (5.2%)	17 (15.7%)	0.0012	15 (5.0%)	14 (16.9%)	0.001^2^	2 (7.4%)	3 (12.5%)	0.656^1^
**Smoking prior to pregnancy**	46 (15.4%)	19 (19.0%)	0.3972	42 (15.5%)	16 (21.3%)	0.231^2^	4 (14.8%)	3 (12.5%)	0.811^1^
**Heredity of diabetes mellitus**	0 (0%)	8 (7.2%)	<0.0011	0 (0%)	4 (4.8%)	0.002^1^	0 (0%)	4 (14.8%)	0.048^1^
**Body Mass Index (BMI) prior to pregnancy** (kg/m2)									
Mean, (± SD)	23.1 (3.5)	26.2 (5.7)	<0.001^3^	23.2 (3.6)	26.2 (5.5)	<0.001^3^	22.3 (2.2)	26.6 (6.4)	0.002^3^
Median, (25th – 75th percentile)	22.6 (20.7-24.2)	25.1 (21.5-29.8)	<0.001^4^	22.6 (20.8 – 24.3)	25.1 (21.4 - 29.8)	<0.001^4^	21.9 (20.3 – 23.7)	26.3 (21.5 – 30.1)	0.267^4^
Min - max	17.2 – 44.9	17.3 – 44.1		17.2 – 44.9	17.3 – 44.1		18.7 – 27.0	18.1 – 43.8	
**Body Mass Index (BMI) classification** ^**5**^ **prior to pregnancy**									
Underweight, BMI <18.49	9 (2.8%)	3 (2.8%)	<0.001^2^	9 (3.1%)	2 (2.4%)	<0.001^2^	0	1 (4.0%)	0.004^2^
Normal weight, BMI 18.50-24.99	251 (77.7%)	47 (43.5%)		227 (76.9%)	36 (43.9%)		23 (85.2%)	10 (40.0%)	
Overweight, BMI 25.00-29.99	48 (14.9%)	32 (29.6%)		44 (14.9%)	24 (29.3%)		4 (14.8%)	8 (32.0%)	
Obesity, BMI ≥30.00	15 (4.6%)	26 (24.1%)		15 (5.1%)	20 (24.4%)		0	6 (24.0%)	
**Physical activity prior to pregnancy**									
No regular physical activity	148 (45.1%)	61 (57.0%)	0.033^2^	134 (44.7%)	40 (49.4%)	0.450^2^	13 (48.1%)	20 (80.0%)	0.0172
**Status During Index Pregnancy 2005**									
**Age**									
Yrs (± SD)	31.0 (4.3)	33.5 (5.0)	<0.001^3^	31.0 (±4.3)	33.4 (4.7)	<0.001^3^	30.7 (4.8)	33.7 (6.2)	0.040^3^
Educational level									
Compulsory school	16 (4.8%)	7 (6.4%)	0.655^2^	12 (4.0%)	2 (2.4%)	0.675^2^	4 (13.8%)	5 (19.2%)	0.647^2^
High school/Folk high school	134 (40.4%)	47 (43.1%)		126 (41.6%)	38 (45.8%)		8 (27.6%)	9 (34.6%)	
University	182 (54.8%)	55 (50.5%)		165 (54.5%)	43 (51.8%)		17 (58.6%)	12 (46.2%)	
**Parity**									
Mean (± SD)	1.3 (0.8)	1.3 (1.3)	0.757^3^	1.3 (0.8)	1.3 (1.1)	0.746^3^	1.4 (1.0)	1.3 (1.7)	0.949^3^
Median, (25th – 75th percentile)	1.0 (1.0 – 2.0)	1.0 (1.0 – 2.0)	0.924^4^	1.0 (1.0 – 2.0)	1.0 (1.0- 2.0)	0.810^4^	1.0 (1.0 – 2.0)	2.0 (1.0 – 3.0)	0.622^4^
Min – Max	0 - 8	0 - 6		0 - 8	0 - 5		0 - 4	0 - 6	
**Physical activity during pregnancy**									
No regular physical activity	178 (55.1%)	69 (63.9%)	0.110^2^	158 (53.4%)	48 (60.0%)	0.291^2^	19 (73.1%)	20 (74.1%)	0.934^2^
**Weight gain during pregnancy (kilograms)**									
Mean (±SD)	14.8 (6.7)	12.8 (7.8)	0.014^3^	14.8 (6.8)	12.2 (7.6)	0.004^3^	14.1 (6.0)	14.4 (8.0)	0.863^3^

^1^Fishers’ test, ^2^Chi2-test, ^3^Student’s *t*-test, ^4^Mann–Whitney test, ^5^Definitions according to The World Health Organization (http://apps.who.int/bmi/index.jsp?introPage=intro_3.html).

### Non-participants

Using available MBR data, the characteristics of the non-participants (n = 429) were compared with those of the participants (n = 444). The non-participants were younger (29.7 yrs., SD ± 5.5) than participants (31.1 yrs., SD ± 4.7) (p < 0.001), and had more children (2.1 children, SD ± 1.2) than participants (1.9 children, SD ± 0.9) (p = 0.027). No significant differences were observed between non-participants and participants regarding gestational age at birth or marital status. Furthermore, no significant differences were seen in the participation rates between women with normal pregnancies, women with diet-treated GDM and women with insulin-treated GDM. Unfortunately, no register data were available that identified ethnic origin.

### Present lifestyle four years after pregnancy

Reported present lifestyle four years after index pregnancy is shown in Table 2. Regarding Swedish snuff (i.e. oral use of wet tobacco), 7.2% of all participants reported regular use of the substance with no differences between groups. No differences were seen among groups regarding smoking; 18.3% of all participants reported smoking.

**Table 2:** **Life style in participants four years after index pregnancy for women with and without previous GDM and born in or outside Sweden**

	**Pregnancy without previous GDM**	**Pregnancy with previousGDM**	**P-value**	**Participants born in Sweden without GDM**	**Participants born in Sweden with GDM**	**P-value**	**Participants born outside Sweden without GDM**	**Participants born outside Sweden with GDM**	**P-value**
**Present status**	**(N = 321)**	**(N = 107)**		**(N = 293)**	**(N = 81)**		**(n = 27)**	**(N = 25)**	
**Pregnant at follow up** (excluded from calculations)	10	4		8	2		2	2	
**Smoking**									
On daily basis/ Occasionally	60 (18.7%)	21 (18.7%)	1.000^1^	51 (17.4%)	16 (19.8%)	0.626^1^	9 (33.3%)	4 (16.0%)	0.205^2^
Never	261 (81.3%)	90 (81.3%)		242 (82.6%)	65 (80.2%)		18 (66.7%)	21 (84.0%)	
**Alcohol habits**									
Never	45 (14.0%)	24 (22.4%)	0.007^1^	35 (11.9%)	10 (12.3%)	0.119^1^	10 (37.0%)	14 (56.0%)	0.035^1^
≤ once a month	93 (29.0%)	43 (40.2%)		90 (30.7%)	36 (44.4%)		3 (11.1%)	7 (28.0%)	
2-4 times/ month	142 (44.2%)	27 (25.2%)		131 (44.7%)	25 (30.9%)		10 (37.0%)	2 (8.0%)	
2-3 times/ week	38 (11.8%)	12 (11.2%)		34 (11.6%)	10 (12.0%)		4 (14.8%)	1 (4.0%)	
≥4 times/week	3 (0.9%)	1 (0.9%)		3 (1.0%)	0		0	1 (4.0%)	
**Units alcohol at each drinking occasion**									
1-2 units	184 (67.2%)	61 (74.4%)	0.215^1^	171 (66.5%)	51 (71.8%)	0.399^1^	12 (75.0%)	9 (90.0%)	0.617^2^
≥3 units	90 (32.8%)	21 (25.6%)		86 (33.5%)	20 (28.2%)		4 (25.0%)	1 (10.0%)	
**Eating habits**									
3 main courses/day^5^	262 (82.4%)	78 (72.9%)	0.034^1^	245 (84.5%)	60 (74.1%)	0.030^1^	16 (59.3%)	17 (68.0%)	0.512^2^
Irregular habits^6^	56 (17.6%)	29 (27.1%)		45 (15.5%)	21 (25.9%)		11 (40.7%)	8 (32.0%)	
**Amount of fruit and vegetables/day**									
% of recommended amount, mean (±SD)	63.1% (±25.2)	61.1% (±25.1)	0.494^3^	63.5% (±25.1)	60.4% (±23.0)	0.308^3^	56.4% (±25.1)	62.1% (±31.0)	0.471^3^
**Reached recommended amount of fruit and vegetables/day** ^**7**^									
Yes	20 (6.3%)	9 (8.4%)	0.451^1^	18 (6.2%)	3 (3.7%)	0.586^2^	1 (3.8%)	5 (20.0%)	0.099^2^
No	298 (93.7%)	98 (91.6%)		273 (93.8%)	78 (96.3%)		25 (96.2%)	20 (80.0%)	
**Physical activity on regular basis**									
Yes	197 (63.3%)	54 (53.5%)	0.077^1^	186 (64.6%)	47 (60.3%)	0.481^1^	11 (50.0%)	6 (27.3%)	0.215^1^
No	114 (36.7%)	47 (46.5%)		102 (35.4%)	31 (39.7%)		11 (50.0%)	16 (72.7%)	
**Frequency of physical activity/week** ^**8**^									
No of times/week,									
Mean (±SD)	2.6 (1.6)	2.6 (1.8)	0.759^3^	2.6 (1.5)	2.8 (1.8)	0.490^3^	2.6 (1.3)	2.7 (1.4)	0.910^3^
Median, 25th -75th percentile	2.0 (1.2 - 3.0)	2.0 (1.2 - 3.4)	0.922^4^	2.0 (1.5 – 3.0)	2.0 (1.5 – 3.5)	0.919^4^	2.5 (1.0 – 4.0)	2.5 (1.75 – 3.5)	1.000^4^
**Intensity of performed physical activity** ^**8**^									
High	57 (29.1%)	5 (9.6%)	0.006^1^	55 (29.7%)	4 (8.5%)	0.007^1^	2 (18.2%)	1 (25.0%)	0.774^2^
Moderate	102 (52.0%)	30 (57.7%)		97 (52.4%)	29 (61.7%)		5 (45.5%)	1 (25.0%)	
Low	37 (18.9%)	17 (32.7%)		33 (17.8%)	14 (29.8%)		4 (36.4%)	2 (50.0%)	
**Reached the recommended level of physical activity** ^**8**^									
Yes^9^	65 (33.0%)	10 (19.2%)	0.062^2^	61 (32.8%)	10 (21.3%)	0.156^2^	4 (36.4%)	0	0.516^2^
No	132 (67.0%)	42 (80.8%)		125 (67.2%)	37 (78.7%)		7 (63.6%)	4 (100%)	

^1^Chi2-test, ^2^Fishers’ test, ^3^Student’s *t*-test, ^4^Mann–Whitney test, ^5^Breakfast, lunch and dinner with and without 1 – 3 snacks, ^6^Skipping courses; ie. reporting irregular eating habits, such as 1 – 2 main courses/ day or frequent snacking and no main courses, ^7^500 grams of fruit and vegetables/day, ^8^Only participants reporting regular physical activity included in this analysis, ^9^≥ 3 times/week at moderate or high intensity.

**Figure 2:**
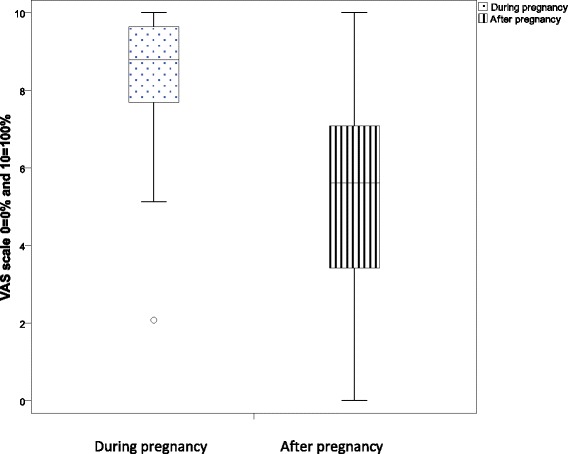
**Boxplot presenting reported compliance with dietary advice during and after pregnancy complicated by gestational diabetes mellitus (GDM).**

Women with a history of GDM reported less compliance to the dietary advice at the time of the follow-up compared to during pregnancy (p < 0.001) (Figure 2). On average, participants reached 62.5% of the recommended daily amount of fruit and vegetables, which is equivalent to approximately 310 grams each day. The national recommendation of daily intake of 500 grams of fruit and vegetables was reached by 7.3% of all participants. No differences in reaching recommendations were seen between women with a history of normal pregnancy and women with a history of GDM. A larger proportion of the women with a history of GDM reported drinking alcohol less than once a month or never. However, this difference was only seen among participants born outside Sweden. Women born in Sweden with a history of GDM, had more often irregular eating habits (i.e. skipping meals or frequently eating snacks) compared to women born in Sweden with normal pregnancy (p = 0.030). This difference was not found for women born outside Sweden.

Of all participants, 30.9% reported being physically active on a regular basis at all three time periods (prior to pregnancy, during pregnancy, and present status). Significantly fewer women with a history of GDM were physically active at all three time periods compared to women with a history of normal pregnancy (21.8% vs. 33.9%, p = 0.022). The risk of being inactive at all three time periods was more than doubled among women born outside Sweden compared to women born in Sweden (OR 2.30, 95% CI: 1.21 – 4.36).

Further, 39.1% of all participants were inactive at the follow-up with no differences between women with a history of normal pregnancy and women with a history of GDM. A significantly larger proportion of women born outside Sweden were inactive at the time of the follow-up compared to women born in Sweden (60.4% vs. 36.3%, p = 0.001). Among women being physically active on a regular basis at the time of the follow-up, women with a history of GDM exercised at low intensity to further extent than did women with a history of normal pregnancy. This difference in intensity of PA was not seen among women born outside Sweden.

### Health status three to four years after pregnancy

Health status among participants four years after index pregnancy is presented in Table 3. In total, the participants reported an increase of body weight at the follow-up, and no differences were found between groups. However, the individual changes of body weight ranged between losing 47.0 kg and gaining 35.0 kg at the follow-up compared to prior the pregnancy.

**Table 3:** **Health status in participants four years after index pregnancy presented for women with normal pregnancy and women with a history of gestational diabetes mellitus (GDM) and as born in and outside Sweden born in or outside Sweden**

	**Normal pregnancy 2005**	**Pregnancy with GDM 2005**	**P-value**	**Participants born in Sweden with normal pregnancy 2005**	**Participants born in Sweden with GDM 2005**	**P-value**	**Participants born outside Sweden with normal pregnancy 2005**	**Participants born outside Sweden with GDM 2005**	**P-value**
**Present status**	**(N = 321)**	**(N = 107)**		**(N = 293)**	**(N = 81)**		**(N = 27)**	**(N = 25)**	
**Pregnant or missing pregnancy status at follow up** (excluded from calculations)	12	4		10	2		2	2	
**Overt diabetes mellitus**									
Yes	0	19 (17.8%)	<0.001^1^	0	13 (16.0%)	<0.001^1^	0	6 (24.0%)	0.009^1^
No	321 (100%)	88 (82.2%)		293 (100%)	68 (84.0%)		27 (100%)	19 (76.0%)	
**Self-rated health at follow-up**									
Excellent, very good, good	291 (92.7%)	80 (80.0%)	<0.001^2^	267 (92.4%)	66 (88.0%)	0.225^2^	23 (95.8%)	13 (54.2%)	0.002^1^
Fair, poor	23 (7.3%)	20 (20.0%)		22 (7.6%)	9 (12.0%)		1 (4.2%)	11 (45.8%)	
**Body weight at follow-up (kilograms)**									
Mean (± SD)	66.5 (11.7)	73.1 (16.7)	<0.001^3^	66.9 (12.0)	73.5 (16.8)	<0.001^3^	62.8 (8.0)	72.1 (16.8)	0.013^3^
Median (25th – 75th percentile)	65.0 (58.0-71.0)	71.0 (60.8–80.5)	0.003^4^	65.0 (58.0–72.0)	73.0 (60.0–81.5)	0.010^4^	65.0 (54.0–68.0)	69.0 (62.0– 81.2)	0.125^4^
(Min –max)	43.0 – 116.0	42.0 – 122.0		43.0 – 116.0	42.0 – 122.0		50.0 – 80.0	48.0 – 109.0	
**BMI at follow-up, (kg/m2)**									
Underweight, (<18.49)	4 (1.3%)	2 (1.9%)	<0.001^2^	4 (1.4%)	2 (2.5%)	<0.001^2^	0	0	0.013
Normal weight, (18.5-24.99)	229 (72.0%)	48 (45.7%)		208 (71.7%)	37 (45.7%)		20 (74.1%)	10 (41.7%)	
Overweight, (25.0-29.99)	62 (19.5%)	26 (24.8%)		56 (19.3%)	21 (25.9%)		6 (22.2%)	6 (25.0%)	
Obesity, (≥30.0)	23 (7.2%)	29 (27.6%)		22 (7.6%)	21 (25.9%)		1 (3.7%)	8 (33.3%)	
**Weight change (pre-pregnancy to follow-up, kilograms)**									
Mean (± SD)	+1.6 (5.7)	+1.3 (8.9)	0.736^3^	+1.5 (5.8)	+0.7 (9.4)	0.319^3^	+2.2 (5.4)	+3.6 (6.5)	0.389^3^
Median (25th – 75th percentile)	+1.0 (−1.0 - +3.2)	+1.0 (−2.0 - +6.0)	0.736^4^	+1.0 (−1.0- +3.0)	+1.0 (−2.0- +4.8)	0.931^4^	+1.5 (−1.2- +5.0)	+3.0 (0 - +8.0)	0.328^4^
(Min –max)	−20.0 - + 35.0	−47.0 - + 20.0		−20.0 - + 35.0	−47.0 - + 22.0		−7.0 - + 16.0	−10.0 - + 16.0	
**Sick leave for more than one week due to health problems** ^**5**^									
Yes	60 (18.8%)	35 (33.3%)	0.002^2^	52 (17.7%)	27 (33.3%)	0.002^2^	8 (29.6%)	8 (33.3%)	0.776^2^
No	260 (81.2%)	70 (66.7%)		241 (82.3%)	54 (66.7%)		19 (70.4%)	16 (66.7%)	
**Medication on regular basis** ^**6**^									
Yes	52 (16.2%)	45 (42.1%)	<0.001^2^	48 (16.4%)	34 (42.0%)	<0.001^2^	4 (14.8%)	11 (44.0%)	0.032^1^
No	269 (83.8%)	62 (57.9%)		245 (83.6%)	47 (58.0%)		23 (85.2%)	14 (56.0%)	

^1^Fisher’s Exact Test, ^2^Chi2test, ^3^Student’s *T*-test, ^4^Mann Whitney, ^5^Sick leave for longer period than one week requires a medical certificate issued by a physician, ^6^Hormonal contraceptives were excluded in the analysis.

In total, 22.7% of all participants reported using medication on a regular basis; 42.3% of the women with a history of GDM compared to 16.2% of women with normal pregnancy (p < 0.001). The reported conditions requiring medication were asthma/allergy (6.8%), depression (5.6%), diabetes (3.8%), thyroid conditions (3.6%), pain problems (2.5%), hypertension (0.5%) and other conditions in 1.4% of all participants. Women with a history of GDM reported using analgesics (6.7% vs. 1.5%, p = 0.007) and thyroid medication to a higher extent (11.7% vs. 1.8%, p < 0.001) than did women with a history of normal pregnancy. Only women with a history of GDM used medication for diabetes mellitus and no differences between groups were found regarding medication for asthma/allergy or depression.

At the time of the follow – up, 19 participants reported a diagnosis of diabetes. All participants with overt diabetes at the follow – up had GDM in their index pregnancy and 16 of them (84.2%) had been treated with insulin during pregnancy.

### Fair/ poor SRH, lifestyle, and health status

Forty-three women (9.7%) reported fair or poor SRH at the follow-up. The associations of fair/poor SRH in relation to present lifestyle and health status are presented in Table 4. Reporting fair/poor SRH was related to being born outside Sweden and to lifestyle factors such as irregular eating habits and no regular PA, as well as being overweight or obese, and regular use of medication. In the univariate analysis, an index pregnancy complicated by GDM more than tripled (OR 3.13, 95% CI: 1.64 – 5.97) the risk of reporting fair/poor SRH at the follow-up. However, adjusting for the effects of being born in or outside Sweden, present BMI, sick leave for more than one week since childbirth 2005, medical treatment on regular basis and overt diabetes, we found that this association did not remain significant. Furthermore, being diagnosed with overt diabetes mellitus at the time of the follow-up was not associated with fair/poor SRH neither in the univariate nor multivariate analysis.

**Table 4:** **Odds ratios (ORs) and their 95% confidence intervals (CI) for fair/poor self-rated health (SRH) for women with normal pregnancy and women with a history of gestational diabetes mellitus (GDM) in relation to life style and health status four years after index pregnancy in univariate and stepwise multivariate logistic regression analyses**

**Present Life Style**	**Fair/poor SRH (n = 43) No. (%)**	**Good SRH (n = 386) No. (%)**	**Univariate OR for poor SRH**	**95% CI**	Multivariate OR* for poor SRH	**95% CI**			**Multivariate OR** ^**#**^ **for poor SRH**	**95% CI**
**Smoking 2009**										
No	32 (74.4%)	318 (82.4%)	1.0							
Yes	11 (25.6%)	68 (17.6%)	0.62	0.30 – 1.29						
**Alcohol 2009**										
Never/≤ once/month	31 (72.1%)	180 (46.6%)	1.0							
≥2-4 times/month	12 (27.9%)	206 (53.4%)	0.34	0.17 – 0.68	0.46	0.22-0.95			0.81	0.34-1.91
Eating habits 2009										
3 main courses	6 (60.5%)	314 (82.0%)	1.0							
Irregular meals	17 (39.5%)	69 (18.0%)	2.98	1.53 – 5.78	2.84	1.36-5.79			2.82	1.22-6.53
**Physical activity on regular basis 2009**										
Yes	13 (33.3%)	240 (63.8%)	1.0							
No	26 (66.7%)	136 (36.2%)	3.53	1.76 – 7.10	3.01	1.47-6.15			2.86	1.26-6.49
**Present health status**							Multivariate OR* for poor SRH	95% CI		
**Pregnancy 2005**										
Normal	23 (53.5%)	302 (78.2%)	1.0							
GDM	20 (46.5%)	84 (21.8%)	3.13	1.64 – 5.97			0.97	0.43-2.19	0.80	0.33-1.98
**Born outside Sweden**										
No	31 (72.1%)	344 (89.6%)	1.0							
Yes	12 (27.9%)	40 (10.4%)	3.33	1.58 – 7.00			2.61	1.00- 6.77	2.21	0.78-6.24
**Body Mass Index 2009**										
≤24.99	9 (22.0%)	268 (72.6%)	1.0							
25.0 – 29.99	15 (36.6%)	69 (18.7%)	6.47	2.72 – 15.42			5.25	2.14- 12.86	5.95	2.24-15.83
≥30.0	17 (41.5%)	32 (8.7%)	15.82	6.51 – 38.42			9.94	3.76- 26.29	11.40	3.78-34.34
**Sick leave for more than one week since 2005**										
No	23 (54.8%)	305 (79.4%)	1.0							
Yes	19 (45.2%)	79 (20.6%)	3.19	1.66 – 6.15			1.80	0.84- 3.85	1.12	0.47-2.65
**Medical treatment on regular basis 2009**										
No	20 (46.5%)	314 (81.3%)	1.0							
Yes	23 (53.5%)	72 (18.7%)	5.02	2.61 – 9.62			2.97	1.39-6.37	3.18	1.38-7.36
**Diabetes diagnosis 2009**										
No	40 (93.0%)	371 (96.1%)	1.0							
Yes	3 (7.0%)	15 (3.9%)	1.86	0.52 – 6.68						

*Significant variables in the univariate analysis of life style and health status respectively were entered in a stepwise manner. Only last step of stepwise multiple regression analysis is presented. ^#^Variables included in the final analysis: alcohol 2009, eating habits 2009, physical activity on regular basis 2009, pregnancy 2005, born outside Sweden, Body Mass Index 2009, and medical treatment on regular basis 2009.

## Discussion

This study found significant differences between women with normal pregnancy/delivery and women with a pregnancy complicated by GDM with respect to lifestyle and health status approximately four years after childbirth. Furthermore, the study revealed that irregular eating habits, lack of regular PA, being overweight or obese, as well as being on regular medication were all associated with poorer SRH at the follow-up. However, being diagnosed with GDM at the index pregnancy or with overt diabetes mellitus at the follow-up showed no association with fair/poor SRH at the time of the follow-up.

### Lifestyle four years after pregnancy

Previous research suggests that a diet comprising high quantities of fruits and vegetables, whole grains, poultry, and fish together with less consumption of starchy and processed foods, red meat, and sugar-sweetened drinks may delay the development of type 2 diabetes [20]. In our study, Swedish born women with a history of GDM reported having more irregular meals than women with a history of normal pregnancy, a fact that is of concern. Additionally, irregular eating habits showed a significant association with poorer SRH. Irregular eating habits in relation to development of diabetes are sparsely studied; however, it has been shown that older women with irregular breakfast eating habits are at increased risk for development of diabetes [21]. Further, there is an increased risk of metabolic syndrome when irregular energy intake during breakfast and in between meals is reported [22]. Elsewhere, limited consumptions of fruits and vegetables among women with a history of GDM are also reported. In an Australian survey of postpartum eating habits, 5% of participants with previous GDM consume the recommended amount of vegetables per day and 44% reach the recommended amount of fruit [23].

In a Canadian prospective observational cohort study, women with a history of GDM reported lower pre-pregnancy leisure and sport activity than a control group of women without GDM. One year after childbirth, no difference between groups regarding leisure or sports activity is reported, indicating that women with a history of GDM increase their postpartum PA in the year following birth [24]. A similar pattern was found in our study; the significant pre-pregnancy difference disappeared during pregnancy and after childbirth, which might indicate increased PA among women with a history of GDM. Despite this observed increase in PA, almost half of the participants with previous GDM in our study did not perform regular PA, a finding that differs from some international studies. An Australian study of women with a history of GDM show that 26.5% have a sedentary lifestyle [25]. Our findings are more similar to those reported in a Danish follow-up study of 121 women with previous GDM, where 36-40% of women do not exercise after childbirth [10]. As previously mentioned, a Cochrane review shows that the incidence of type 2 diabetes mellitus in high-risk populations may be decreased by interventions that increase PA and improve diet [8]. Despite some improvements in performance of PA noticed in our study, most women with previous GDM do not seem to change their lifestyle according to recommendations.

### Health status four years after pregnancy

Internationally, differences in self-reported health status for women with a history of normal pregnancy and women with a history of GDM have been reported. In women with previous GDM, a prevalence of 12 – 13.8% fair/poor SRH are reported compared to 7 – 9.3% in women with no GDM [26,27]. In our study, 20% of women with a history of GDM reported fair/poor SRH. However, when adjusting for other risk factors, GDM at index pregnancy was no longer associated with poorer SRH. Being born outside Sweden was found to be associated with poorer SRH; this finding is in agreement with other studies comparing SRH of immigrants and Swedish born individuals [28,29]. However, the SRH status may be affected by other life events mediating the findings. In a qualitative study, women evaluate their whole life situation when answering questions on their SRH, i.e. capturing physical symptoms and emotional problems affecting daily life as well as family functioning, relationship with partner and balancing motherhood and work. Events relating to childbirth were not included in the responses one year after childbirth [30]. Also, poor SRH in pregnancy and six months after childbirth reveal associations with lower levels of social support and friendship network [31].

Previous studies indicate that the cumulative incidence of type 2 diabetes mellitus rapidly increases within the first five years after pregnancy [6]. Other Swedish studies report 11% overt diabetes among women with a history of GDM one to two years postpartum [32] and 30% five years postpartum [33]. Our findings correspond to these results. However, there was no information regarding what type of overt diabetes the women had developed. Hence, there might be biological explanations mediating the development of diabetes not accounted for in this study.

Our findings indicated sub-optimal levels of PA and intake of fruit and vegetables in women with a history of GDM as well as in women with normal pregnancy. Furthermore, irregular eating habits, no regular PA, being overweight or obese and on regular medication showed associations with poorer SRH. Health care providers and societal organizations should initiate counselling and interventions regarding diet and PA for all women postpartum as to remain healthy or improve future health. Further research may address how interventions and counselling aimed to increase PA and improve intake of fruit and vegetables are experienced, especially among women with a history of GDM.

### Methodological discussion

The number of non-participants might be considered high in this study; however, the initial sample was based on a random national register selection. A Cochrane review studying strategies to improve the response rate in questionnaires, reveal that the response rate is higher if, for example, hand-written envelopes and shorter questionnaires are used. Also, the response rate decreases when questions addressing sensitive topics are included [34]; i.e. factors that may have contributed to the eligible participants’ willingness to participate. One reminder was used for this data collection. Additional reminders may have improved the final response rate. Others describe how the response rate increases continuously with up to four reminders in a questionnaire study [35]. Another explanation to the low participation rate may be that the questionnaire was administrated in Swedish, which may have resulted in fewer immigrants participating. The non-participants were significantly younger and had more children; i.e. a situation that may have skewed some results. These limitations should be considered when evaluating the findings of this study.

The NFA suggests a number of questions to be used as an indicator of quality of diet. The quantity of fruits and vegetables is estimated by reporting numbers of servings per month, week, or day of fruit and vegetables respectively [19]. These NFA questions were not used in this survey; instead the respondents were asked to estimate to what extent they reached the recommendation of fruit and vegetables. Therefore, the reported compliance to recommendations of fruit and vegetables may be less valid compared had the NFA indicators of quality of diet been used. Yet, a review found that the VAS is associated with energy intake well enough to be used as a proxy of energy intake. Further, VAS is predicting with reasonable certainty meal initiation and amount consumed [36].

The respondents recalled some issues regarding pregnancy and postpartum approximately four years before this study. Studies of maternal recall of events related to pregnancy and delivery show high accuracy even several years postpartum [37,38], hence recall bias of such events should not be considered a major limitation of our study. However, during the years following the index pregnancy and delivery, some women may have experienced life events not addressed in the questionnaire that might have affected their lifestyle and health status.

The concept of social desirability (i.e. the tendency of individuals to report themselves in coherence with perceived cultural norms) is associated with overestimation of PA [39] as well as underestimation of energy intake, especially in women [40]. This type of bias may have occurred as there were no objective measures of PA and diet compliance in this population-based study. Additionally, few studies have addressed the reliability of long-term recall of health behaviors. Recall of intense PA is more accurate 10 years later than less intense activities [41]. A more accurate recall regarding more intense activities is also seen 15 years later [42]. Hence, there is little reason to believe that recall bias regarding the more intense PA had a major negative impact on the validity of our findings.

The strength of our study includes the detailed answers provided by the participants, who also represent a variety of women covering all geographical regions of the country, factors that may increase the validity of the results. To address the internal validity of the data, a rigorous control of the accuracy of registered data was performed. Despite the limitations described, our findings agreed with previous studies [43], hence we believe that our study may reflect the lifestyle and health status of women with and without a history of GDM living in similar settings.

## Conclusions

These results reveal suboptimal levels of PA, and fruit and vegetable consumption for a sample of women with a history of GDM as well as for women with normal pregnancy approximately four years after index pregnancy. Women with previous GDM seem to increase their PA after childbirth, but still they perform their activity at lower intensity than women with a history of normal pregnancy. Having GDM at index pregnancy or being diagnosed with overt diabetes mellitus at follow-up did not demonstrate associations with poorer SRH four years after delivery.

## Additional file

Additional file 1:
**An overview of potential predictors, outcomes and data source.**
