# Supplementary material for: Lifestyle and health status in a sample of Swedish women four years after pregnancy: a comparison of women with a history of normal pregnancy and women with a history of gestational diabetes mellitus
Source: BMC Pregnancy Childbirth. 2015 Mar 13;15:57. doi: 10.1186/s12884-015-0487-2 (PMC4372034; doi:10.1186/s12884-015-0487-2)
Supplement: Additional file 1: — An overview of potential predictors, outcomes and data source. [file 12884_2015_487_MOESM1_ESM.docx]

Appendix 1. An overview of potential predictors, outcomes and data source

| **OUTCOME** | **VARIABLES USED AS POTENTIAL PREDICTORS** |
| --- | --- |
| **Background characteristics**  Born in Sweden (yes/no)^1^  Normal pregnancy and delivery/ GDM diagnosis in index pregnancy^2^ | Maternal age^2^  Parity before index pregnancy^2^  Marital status (married, co-habiting, single)^2^  Educational level (university, high school, elementary school)^1^  Smoking/use of snuff (yes/no; amount on daily basis)^1^  Alcohol use (yes/no; amount on weekly basis)^1^  Infertility treatment (yes/no)^1^  PCOS diagnosis (yes/no)^1^  Self-reported maternal height and body weight^1^  Self-reported weight gain during pregnancy^1^  Self-reported physical activity before and during pregnancy (frequency per week and intensity)^1^ |
| **Lifestyle after pregnancy**  Born in Sweden (yes/no)^1^  Normal pregnancy and delivery/ GDM diagnosis in index pregnancy^2^ | Pregnant at follow-up (yes/no; gestational weeks)^1^  Smoking/use of snuff (yes/no; amount on daily basis)^1^  Alcohol use (yes/ no; amount on weekly basis)^1^  Eating habits (no of courses per day, main meals and snacks)^1^  Fulfillment of recommended levels of fruits and vegetables, (% of recommendation)^1^  Self-reported physical activity before and during pregnancy (frequency per week and intensity)^1^  For women with history of GDM only:   - Useful advice on GDM regime^1^ - Important advice on GDM regime^1^ - Compliance to received advice during and after pregnancy^1^ |
| **Health status after pregnancy**  Born in Sweden (yes/no)^1^  Normal pregnancy and delivery/ GDM diagnosis in index pregnancy^2^ | Pregnant at follow-up (yes/no, gestational weeks)^1^  Overt diabetes mellitus (yes/no)^1^  Medication on regular basis (yes/no; type of drugs)^1^  Sick leave for more than one week (yes/no)^1^  Self-reported maternal body weight^1^  Self-rated health (excellent, very good, good, fair or poor)^1^ |
| **Self-rated health**  Excellent, very good, good = good health  Fair or poor = poorer health | Variables of lifestyle factors and health status significant in univariate or multivariate analysis are included in the model |

1) Data from questionnaire; 2) Data from Medical Birth Register, MBR. For comparisons of participants and non-participants, the following variables from the MBR were used: maternal age, parity before index pregnancy, gestational age at birth, marital status, and status of pregnancy (normal, diet treated gestational diabetes or insulin-treated gestational diabetes).
